# Multisensor hyperspectral imaging approach for the microchemical analysis of ultramarine blue pigments

**DOI:** 10.1038/s41598-021-04597-7

**Published:** 2022-01-13

**Authors:** M. González-Cabrera, K. Wieland, E. Eitenberger, A. Bleier, L. Brunnbauer, A. Limbeck, H. Hutter, C. Haisch, B. Lendl, A. Domínguez-Vidal, M. J. Ayora-Cañada

**Affiliations:** 1 Department of Physical and Analytical Chemistry, Universidad de Jaén, Campus Las Lagunillas, s/n, 23071 Jaén, Spain; 2 Institute of Chemical Technologies and Analytics, TU Wien, Getreidemarkt 9/164, 1060 Vienna, Austria; 3 Chair of Analytical Chemistry, Technical University of Munich, Elisabeth-Winterhalter-Weg 6, 81377 Munich, Germany

**Keywords:** Analytical chemistry, Characterization and analytical techniques, Imaging techniques, Mass spectrometry, Microscopy, Optical spectroscopy

## Abstract

This work presents a multisensor hyperspectral approach for the characterization of ultramarine blue, a valuable historical pigment, at the microscopic scale combining the information of four analytical techniques at the elemental and molecular levels. The hyperspectral images collected were combined in a single hypercube, where the pixels of the various spectral components are aligned on top of each other. Selected spectral descriptors have been defined to reduce data dimensionality before applying unsupervised chemometric data analysis approaches. Lazurite, responsible for the blue color of the pigment, was detected as the major mineral phase present in synthetic and good quality pigments. Impurities like pyrite were detected in lower quality samples, although the clear identification of other mineral phases with silicate basis was more difficult. There is no correlation between the spatial distribution of the bands arising in the Raman spectra of natural samples in the region 1200–1850 cm^−1^ and any of the transition metals or rare earth elements (REE). With this information, the previous hypothesis (based on bulk analysis) attributing these bands to luminescence emissions due to impurities of these elements must be revised. We propose the consideration of CO_2_ molecules trapped in the cages of the aluminosilicate structure of sodalite-type. Additionally, correlation between certain Raman features and the combined presence of Ca, P, and REE, in particular Nd, was detected for the lowest quality pigment. Our results highlight the usefulness of fusing chemical images obtained via different imaging techniques to obtain relevant information on chemical structure and properties.

## Introduction

Hyperspectral imaging (HSI) is nowadays a commonly employed technique that collects both spatial information (coordinates x and y) and spectral data (λ) of the sample to build the multidimensional hypercube. Thus, it is frequently used for imaging purposes, since it can provide the spatial distribution of chemical components and it can aid to solve the complexity and heterogeneity difficulties of a given sample at a pre-selected area of interest. In the past fifteen years, the study of chemical composition at the micrometer level using hyperspectral spectroscopic imaging techniques has grown exponentially^1,2^. However, in many cases, the information provided by one analytical technique may not be enough to capture the sample’s overall complexity^3^. For this reason, the combined use of complementary techniques on the sample can solve analytical challenges that are unattainable otherwise. This multi-sensing approach involves the need for data fusion such that every pixel of each imaging technique is aligned on top of each other. Thus, multimodal hyperspectral imaging, also known as multisensor hyperspectral imaging (MSHSI)^4,5^ offers clear advantages, particularly when dealing with complex and heterogeneous samples, such as cells^6,7^, tissues^8,9^ or tumours^10,11^. Nevertheless, its use is still limited, probably due to challenges that arise during image fusion of data recorded with different sensing platforms, with problems associated with the fusion that can be linked to both the spatial and spectral character of images^12^. Different analytical techniques highlight different sample information (e.g. elemental composition, chemical bonds, chemical environment, spatial arrangement of functional groups, etc.), which is an advantage from a chemical point of view. However, uncorrelated information might hamper the alignment step when assembling the hyperspectral datacube. Besides, the number of channels and the signal intensity of techniques may be diverse necessitating some kind of balance or scaling that has to be introduced. Another key challenge of this approach includes the spatial congruence that needs to be achieved among images to be fused. This implies that the analytical techniques being combined exhibit comparable lateral resolution. Furthermore, in order to acquire information from the same area of the sample, the applied techniques must be non-destructive for sequential investigation, except for the last technique employed when this feature is not mandatory anymore. After data acquisition, accurate alignment of single imaging areas is crucial. Depending on the available software settings, the acquired images may be of different sizes and exhibit different lateral resolutions. In addition, images might be tilted or mirrored with respect to the microscope visible image. Pre-marked areas on the sample surface—if possible—are advisable to facilitate this task^13^.

In most examples reported in the literature, data fusion is typically limited to datasets acquired by two different techniques^14,15^. With this work, we contribute to the development of multisensor hyperspectral imaging by testing the capabilities of fusing data from four different analytical techniques for the characterization of ultramarine, a pigment of high historical and pictorial interest, at the microscopic level. Thus, Scanning Electron Microscopy-Energy Dispersive X-Ray Spectroscopy (SEM–EDX), Time of Flight Secondary Ion-Mass Spectrometry (ToF–SIMS), Laser Ablation Inductively Coupled Plasma Mass Spectrometry (LA-ICP-MS) and Raman micro-spectroscopy will be used in order to obtain both elemental (including not only elements at high concentrations but also those at trace levels) and molecular information. Ultramarine blue has been an extraordinarily valued pigment since ancient times, particularly in Europe during the Middle Ages and the Renaissance, when it was so scarce that it was more valued than gold^16,17^. Its bright and characteristic blue color is due to the mineral lazurite (Na_8_Al_6_Si_6_O_24_S_n_)^18,19^, an aluminosilicate present in the lapis lazuli semi-precious rock^20^. Owing to the same chemical structure as sodalite, (Na_8_Al_6_Si_6_O_24_Cl_2_)^21,22^, it holds central cavities commonly reported as β-cages^23^. AlO_4_ and SiO_4_ tetrahedra are located within them, composing an interconnected anionic and cationic 3-dimensional network^24^. Among the ions and radicals that have been reported to be trapped in those cages^25^, trisulfur (S_3_^−˙^) is considered to be predominantly responsible for the unique blue tone of the pigment^26,27^. The presence of S_3_^−^ chromophore can be easily detected using Raman spectroscopy, since it has a strong band at 548 cm^−1^ (symmetric stretching), which is resonance enhanced when using radiation from 500 to 700 nm for excitation^28^. In addition, the signal can be further enhanced in presence of silver nanoparticles^29^. With the introduction of synthetic ultramarine versions in the nineteenth century^30^, the distinction between the natural and synthetic pigments became an analytical challenge for authentication issues and the recognition of restoration interventions. Studies focusing on this topic are reported in literature employing several analytical techniques, such as pulsed laser-induced breakdown spectroscopy (LIBS)^31^, UV–VIS-NIR reflectance spectroscopy with fiber optic probes^32^, as well as Raman^28,33^ and FTIR spectroscopy^34,35^. A simple and efficient way to distinguish natural from synthetic ultramarine pigments, working even in a non-invasive way as demonstrated recently by our group^36^, is to consider a group of bands that emerge in the range between 1200 and 2000 cm^−1^ in the Raman spectra of the pigment recorded using 785 nm excitation. These features were first observed by Schmidt et al.^37^, who attributed them not to true Raman scattering bands but to luminescence emissions of accessory minerals carrying transition metal impurities such as vanadium and titanium. However, further investigation is still needed to clarify their origin since this attribution was based on bulk analysis, hence, other elements might just as well be responsible for the observed features. For example, similar narrow-line emissions (showing bandwidths in the order of typical Raman features) have been reported to be characteristic of rare earth elements (REE) with 4f electronic configuration incorporated in crystalline materials^38^. Our previous studies using Raman micro-imaging on ultramarine pigments with different qualities and hues revealed that these supposed luminescence emissions, although being clearly associated with colorless areas, are concentrated in certain “hotspots'', which are not homogeneously distributed^36^. In this context, the use of multimodal chemical imaging combining molecular and elemental information can be a very useful tool to elucidate the origin of these bands and to provide a more detailed knowledge about the characteristics of ultramarine pigments of different qualities. This combined approach also prevents over-interpretation of data due to simultaneous cross-checks with complementary analytical methods which is especially helpful for complex sample compositions.

## Results and discussion

We analysed natural ultramarine blue pigments (NU) obtained from Afghanistan lapis lazuli rocks of different quality and one synthetic pigment (SU). One of the natural samples (NU-1) was purified according to the traditional procedure described by Cennino Cennini^39^ whereas the other two natural pigments (NU-2 and NU-3) were prepared just by grinding the rock and sieving (< 80 µm). Finally, ultramarine ash (NU-A) is the by-product that remains after the extraction of blue particles in the purification process. The samples were prepared in the form of KBr pellets because this allows (a) imaging of the powder on a flat surface without losing the optical focus and (b) examining the same area of each pellet by consecutive application of the different analytical techniques. To do this, the measurement position was marked on the pellet using tweezers. In this way, elemental and molecular information was obtained from each sample according to the following consecutive order: Raman spectroscopy, SEM–EDX, ToF–SIMS, LA-ICP-MS. Regarding ToF–SIMS and LA-ICP-MS measurements, both techniques involve the ablation of a surface layer of the sample. In the case of ToF–SIMS, this is less than one monolayer (< 2.5 Å) whereas for LA-ICP-MS analysis ~ 1.2 µm of material is removed and analyzed. For this reason, the latter one was the last technique applied to the samples under investigation.

### Image alignment and data fusion

**Figure 1:**
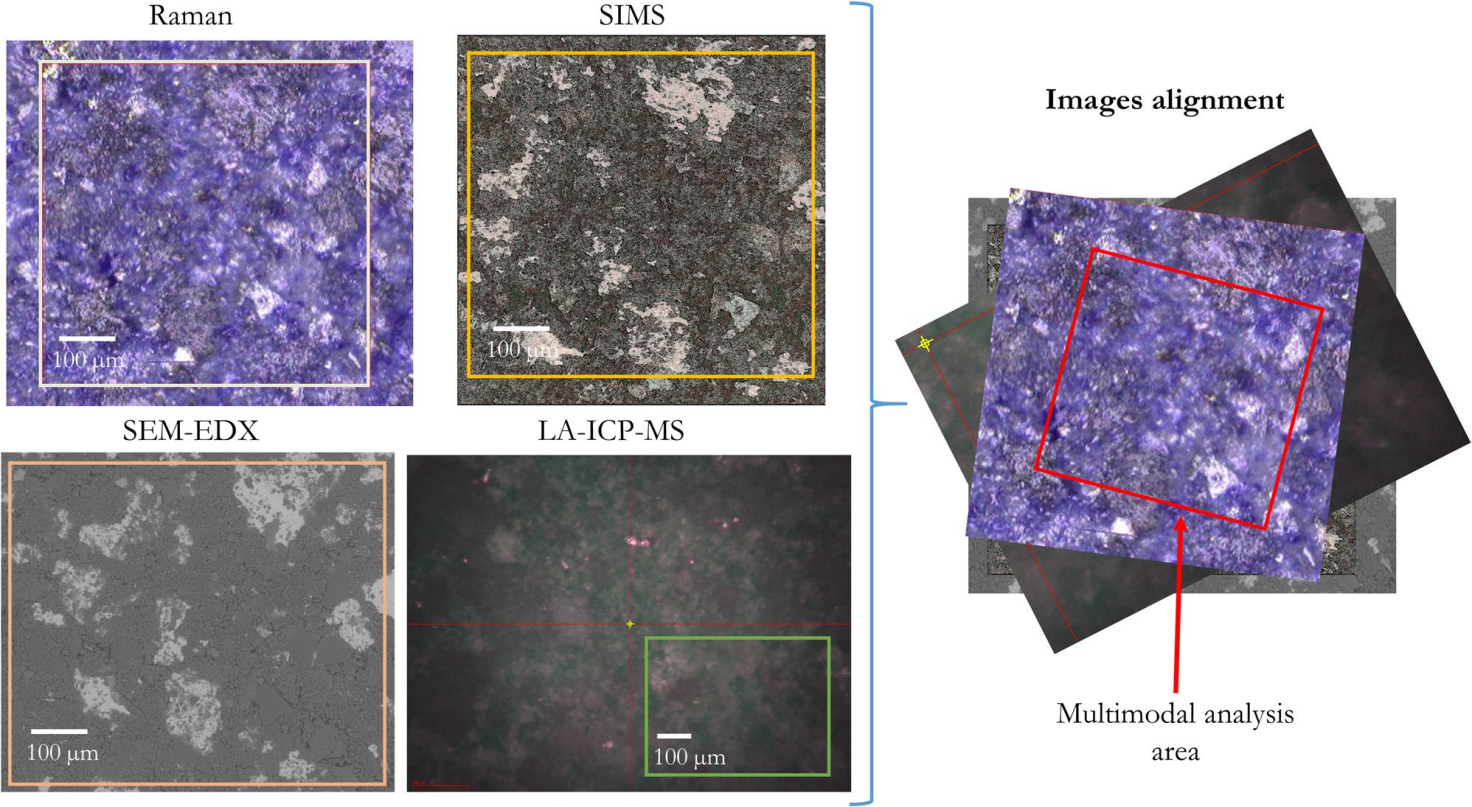
Images of NU-2 sample analyzed with each of the individual techniques (left) and alignment of all datasets via affine transformation (right). Regions not contained within the red square marking overlapping areas of all four imaging techniques were excluded from the multisensor hyperspectral analysis.

Fusion of the individual datasets obtained with the different analytical techniques was performed using the high-resolution image of the sample (SEM image) for all spectral domains. The orientation and basic alignment of the images was first achieved using the tweezer marks on the samples. The fine alignment of the images was performed by an affine transformation, whose parameters were calculated using a bilinear regression model based on matched reference points. The reference points need to be set manually before image transformation. In the case of Raman images, the reference points were manually set according to contrast features in the visible microscope image. Similar features were found in the SEM image facilitating the alignment of EDX and Raman data. The bright KBr areas in the SEM image further aided in the alignment for ToF–SIMS and LA-ICP-MS considering the K signal. The software used for image alignment and processing (ImageLab) allows for the manual selection of reference points in the false color image and in the image plane to which it will be projected on. In our case, between 7 and 10 tie points were manually set for the bilinear affine transformation. The result of the transformed false-color-image superimposed to the reference image is calculated in the same step, which serves as valuable verification for the selected reference points. Thus, individual files containing hyperspectral multisensor datacubes^3^ for each of the samples were created. Figure 1 shows a scheme of the process and an example of this procedure is shown in Supplementary information (Figure S1). In this way, EDX, SIMS, LA-ICP-MS, and Raman spectroscopy datasets were merged into a MSHSI datacube.

### Preliminary data analysis

**Figure 2:**
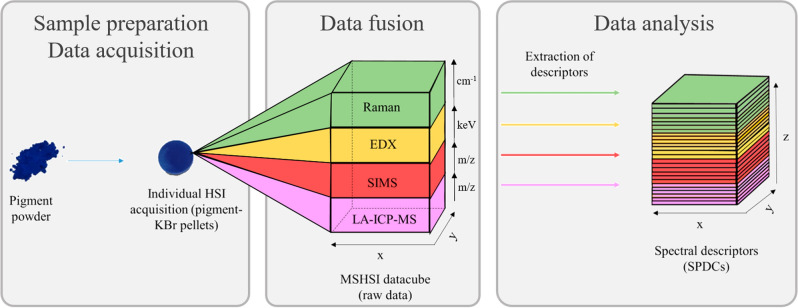
Basic scheme of the hyperspectral imaging acquisition and the methodology for the multisensor datacube acquisition and subsequent selection of spectral descriptors.

A step-by-step approach considering each technique individually was performed on the multisensor hypercube. This permitted detailed analysis of the individual pieces of information gained by each of the analytical techniques and allowed careful selection of the spectral descriptors (SPDCs) for the analysis of the multisensor hypercube. Spectral descriptors were selected to reduce the size of the feature space^13^ (see Fig. 2) and narrow the focus of subsequent data analysis on predefined spectral areas of interest. This step requires detailed sample knowledge to avoid the risk of accidentally excluding crucial information.

First, we checked the distribution maps of the different main and trace elements to evaluate if they were detected with enough sensitivity. Comparison with the synthetic pigment (SU) signals and KBr areas of the pellets aided in this task as they can be used as purity controls (Figure S2, Supplementary information). Thus, when a certain element showed a random distribution (typical of noise) in the images, it was excluded from the analysis. This was the case for Cr (in EDX) and P^+^, Sc^+^, Fe^+^, Si^2+^, Y^+^, Zr^+^, Eu^+^, Pr^+^, and Sm^+^ (in SIMS) Table 1 lists the final SPDCs of each elemental technique included in this study.

**Table 1:** SEM–EDX, SIMS and LA-ICP-MS spectral descriptors included in this study.

Spectral descriptors (SPDCs)					
EDX			SIMS		LA-ICP-MS
Element	Transition	Energy (KeV)	Ion	Mass (m/z)	Isotope
O	Kα	0.525	Na^+^	22.99	^27^Al
Na	Kα	1.041	Mg^+^	23.98	^141^Pr
Mg	Kα	1.254	Al^+^	26.98	^142^Nd
Br	Lα	1.481	Si^+^	27.97	^152^Sm
Al	Kα	1.487	K^+^	38.94	
Si	Kα	1.740	Ca^+^	39.96	
P	Kα	2.014	Ti^+^	47.93	
S	Kα	2.308			
Cl	Kα	2.622			
K	Kα	3.314			
Ca	Kα	3.692			
Ti	Kα	4.511			
V	Kα	4.953			
Mn	Kα	5.889			
Fe	Kα	6.404			

**Figure 3:**
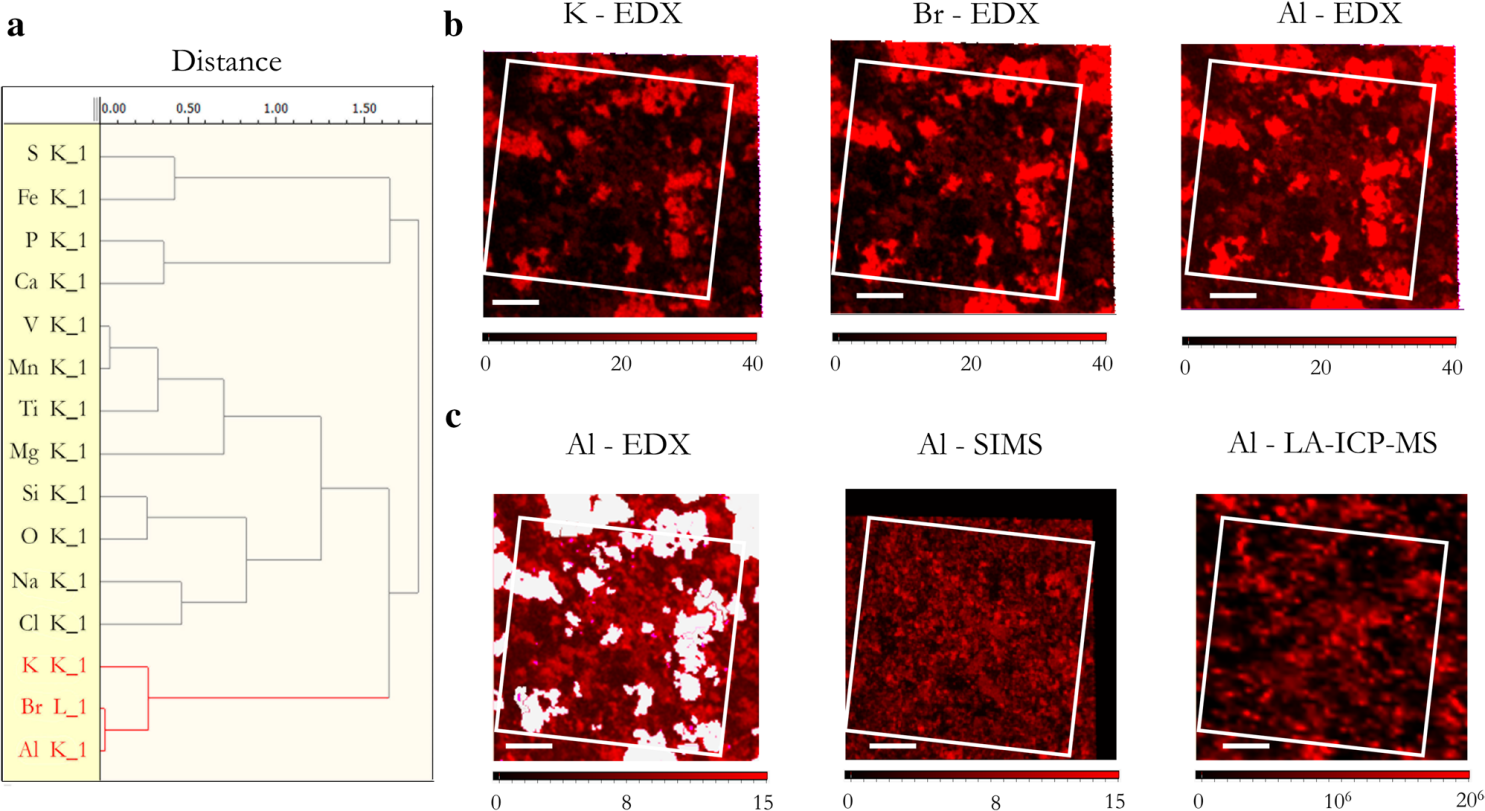
(**a**) Dendrogram obtained from HCA of the first 5 PCs of SEM–EDX SPDCs of NU-A sample. Comparison of spatial distribution for EDX of (**b**) potassium (K), bromide (Br), and aluminium (Al), and (**c**) Al spatial distribution detected by the three elemental techniques with the KBr mask being applied to EDX data (white areas). Common analysis area is marked with a white square. Scale bars correspond to 100 μm.

Additionally, the influence of sample preparation must be considered. In the acquired SEM images (by employing the backscattered electrons mode, BSE mode), dark areas are due to elements with low atomic numbers and correspond mainly to sample particles. On the contrary, the brightest zones, corresponding to elements with high atomic numbers, showed an almost perfect match with the spatial distribution of the cluster formed by K, Br and Al when performing HCA on EDX SPDCs of each sample (see Fig. 3). K and Br are co-localized because they correspond to the KBr used to prepare the pellets and the association of Br and Al is due to the proximity of their respective Lα and Kα peaks in the EDX spectrum. Thus, this cluster mainly represents the heterogeneous distribution of the KBr used in the sample preparation within the total area of measurement. In order to focus data analysis on the pixels corresponding to the pigment samples, excluding the contribution of the KBr matrix, a mask with all pixels of this cluster was created. In the case of natural samples, this mask covers around 25% of the total measured area. However, in the artificial pigment (SU) the mask only covered 9% probably due to the better compaction of the smaller and more homogeneous particle size. Additionally, using the mask, K present in the pigments can be distinguished from that coming from sample preparation and the interference of Br in the identification of Al is significantly reduced. The distributions of Al with EDX (with and without mask), SIMS, and LA-ICP-MS are compared, in Fig. 3b,c, to check the effectiveness of the mask in correcting the interference for Br. As it can be observed, the distribution of Al in EDX without the mask is completely different from those of Al-SIMS and Al-LA-ICP-MS because the most intense signals correspond, in fact, to areas with Br (and K). When applying the mask, these areas are eliminated and the results obtained by the different elemental techniques for Al are more consistent. The differences in depth collection, as commented before, and lateral resolution (leading to a bigger pixel size, in LA-ICP-MS i.e.) justify the discrepancies.

**Figure 4:**
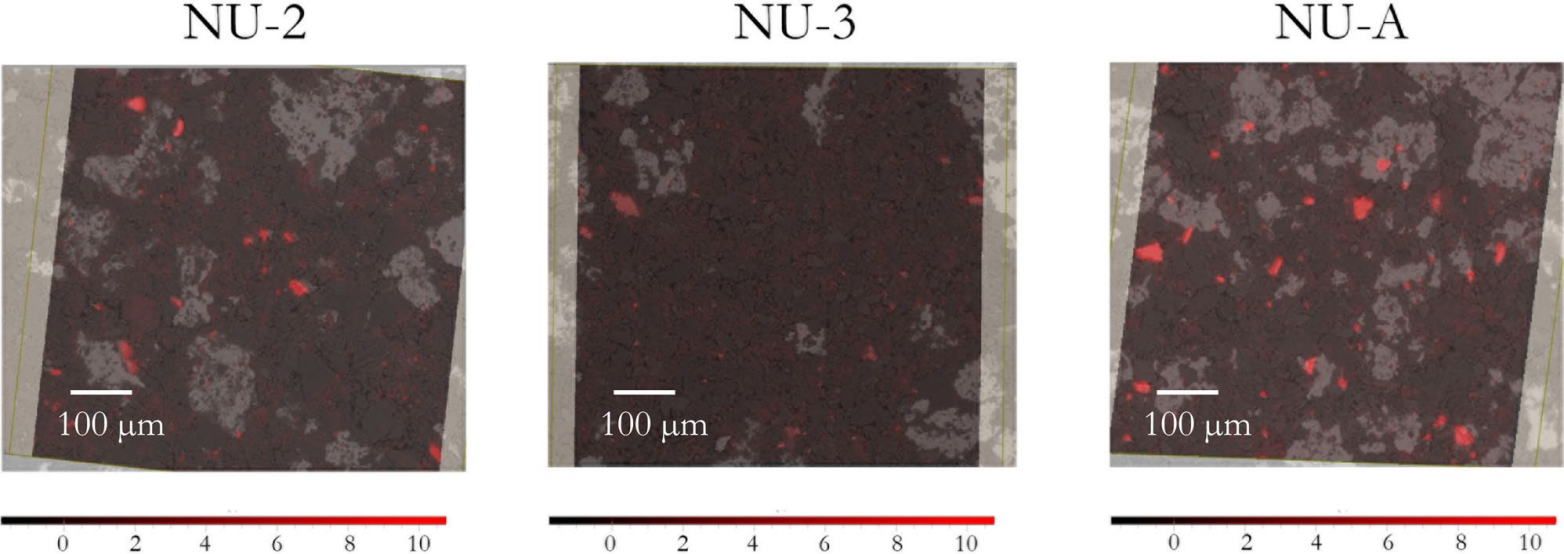
Superposition of Fe-S cluster distribution (red particles) and SEM image of NU-2, NU-3, and NU-A samples.

Further exploration of the dendrograms obtained from HCA of EDX sub-dataset for each pigment shows that Fe and S elemental contributions cluster together (see dendrogram of NU-A in Fig. 3a) in all samples except in the purified one (NU-1) and in the synthetic pigment (SU). These two elements coincide with some of the bright areas of the SEM sample images (see Fig. 4) and can be attributed to pyrite (FeS_2_) grains, a common impurity in natural lapis lazuli^40^. This is in agreement with previous works reporting that pyrite was efficiently removed in the traditional pigment purification process^41^. The NU-A sample, the by-product of the purification process, is clearly enriched in pyrite.

**Figure 5:**
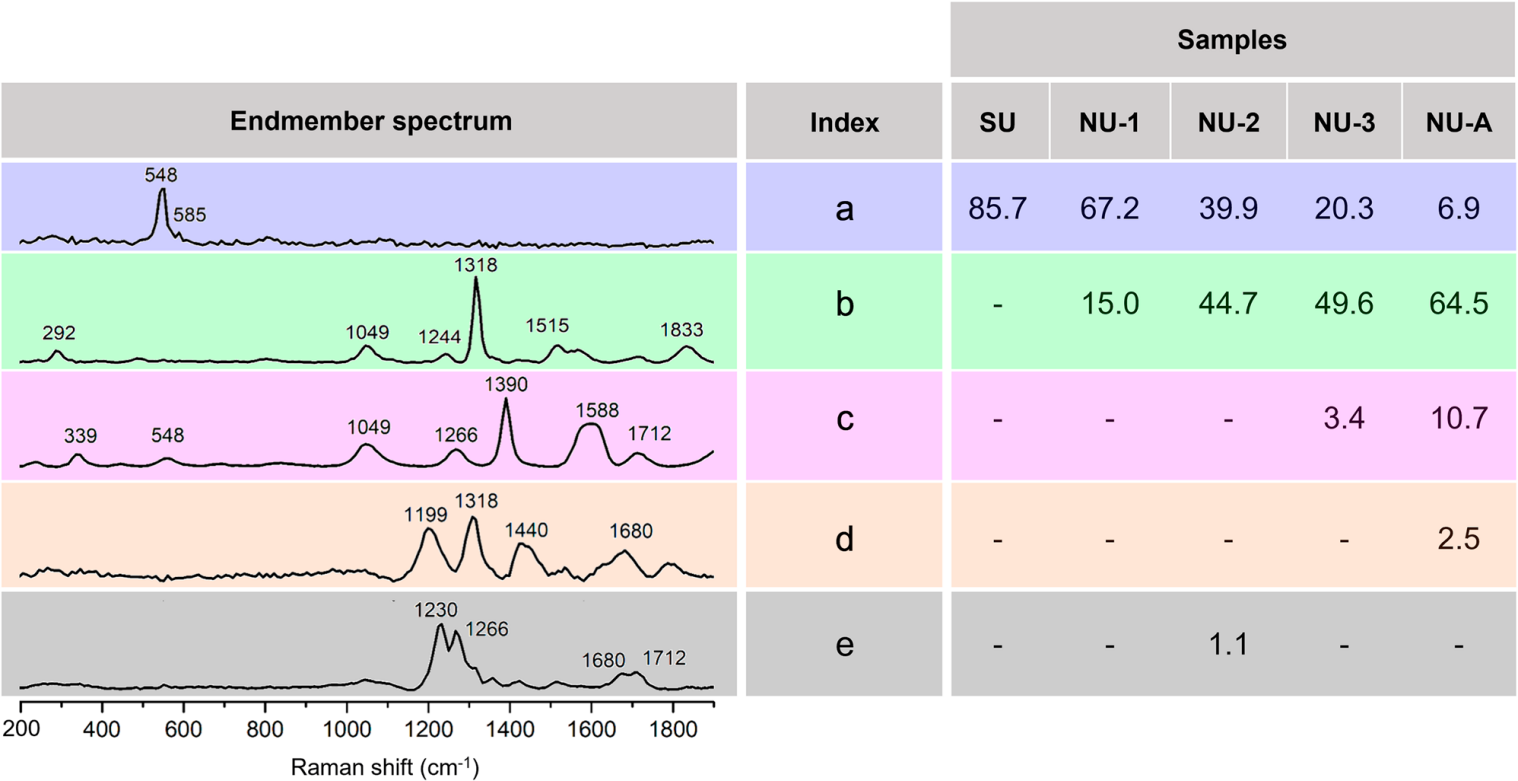
Raman endmembers (**a**–**e**) obtained from the VCA of pigment samples and their respective contributions (%) to the total number of pixels of each sample. The areas of the labelled bands correspond to Raman SPDCs included in the multisensor analysis.

Regarding the Raman sub-dataset, some common bands can be observed in the spectra of the pigments (see Supplementary information, Figure S3). The most prominent one is located at 548 cm^−1^, which is attributed to the symmetric stretching vibration of the S_3_^−^ radical trapped in the lazurite mineral composition. A small shoulder at 585 cm^−1^ is also noticed, especially in the spectra of natural samples, which has been historically associated with both asymmetric stretching of S_3_^−^ radical and symmetric stretching of S_2_^−^ radical^26^. However, it is important to highlight the fact that, depending on the quality of natural pigments, different bands arise in the region between 1200 and 1850 cm^−1^, owing a particular intensity in pixels corresponding to white areas in their visible image, as already reported by González et al.^36^. To select the characteristic Raman bands to be considered as SPDCs for the multisensor analysis, VCA was performed for each sample. This unsupervised technique assumes that the obtained data is composed of a mixture of pure components and extracts their corresponding spectra, so-called “endmembers”. Figure 5 shows the endmembers obtained with good signal-to-noise ratio of the hyperspectral data cube. A rough estimation of their contribution to the total amount of pixels analyzed in each sample was calculated after dichotomization of the image by applying a threshold of 0.5 to the image histogram (data were scaled to an intensity maximum amplitude of 1). Thus, the percentage of pixels containing each endmember revealed that the contribution of the one associated with the lazurite mineral (marked in blue) diminishes as the quality of the pigment decreases (from 85% in SU to 7% in NU-A). On the contrary, the amount and intensity of the bands appearing between 1200 and 1850 cm^−1^ is higher in less purified samples with four distinct spectral patterns. Endmember b is present in all natural samples, whereas endmembers c–e were only found in medium quality and ultramarine ash samples, although their contribution is below 11% in all cases. According to these results, 15 Raman SPDCs were selected, corresponding to the areas of the bands at 292, 339, 548, 585, 1049, 1199, 1244, 1266, 1318, 1390, 1511, 1588, 1680, 1712 and 1833 cm^−1^.

### Multisensor analysis

Multivariate statistics (PCA and HCA) were applied to the MSHSI datacube using the already selected SPDCs. Great differences between scales and intensity values were observed when dealing with the different techniques. Thus, standardization of the data was necessary to achieve proper scaling before building the 3D-datacube. In addition, the KBr mask was applied to set the focus of the data analysis on the pigment sample and to avoid any influence of sample preparation.

PCA condenses information from a large set of variables into fewer uncorrelated variables (PCs) that retain most of the variance present in the original dataset^1^. Thus, each principal component (PC) is a linear combination of the SPDCs. In the created models, loadings, scores, and bi-plots (superposition of loadings and scores contributions of each PC) were considered. Furthermore, hierarchical cluster analysis (HCA) was performed on the loadings of the PCs selected assuming that SPDCs describing similar chemical species can be linked to each other and would accumulate in subclusters of the HCA.

**Figure 6:**
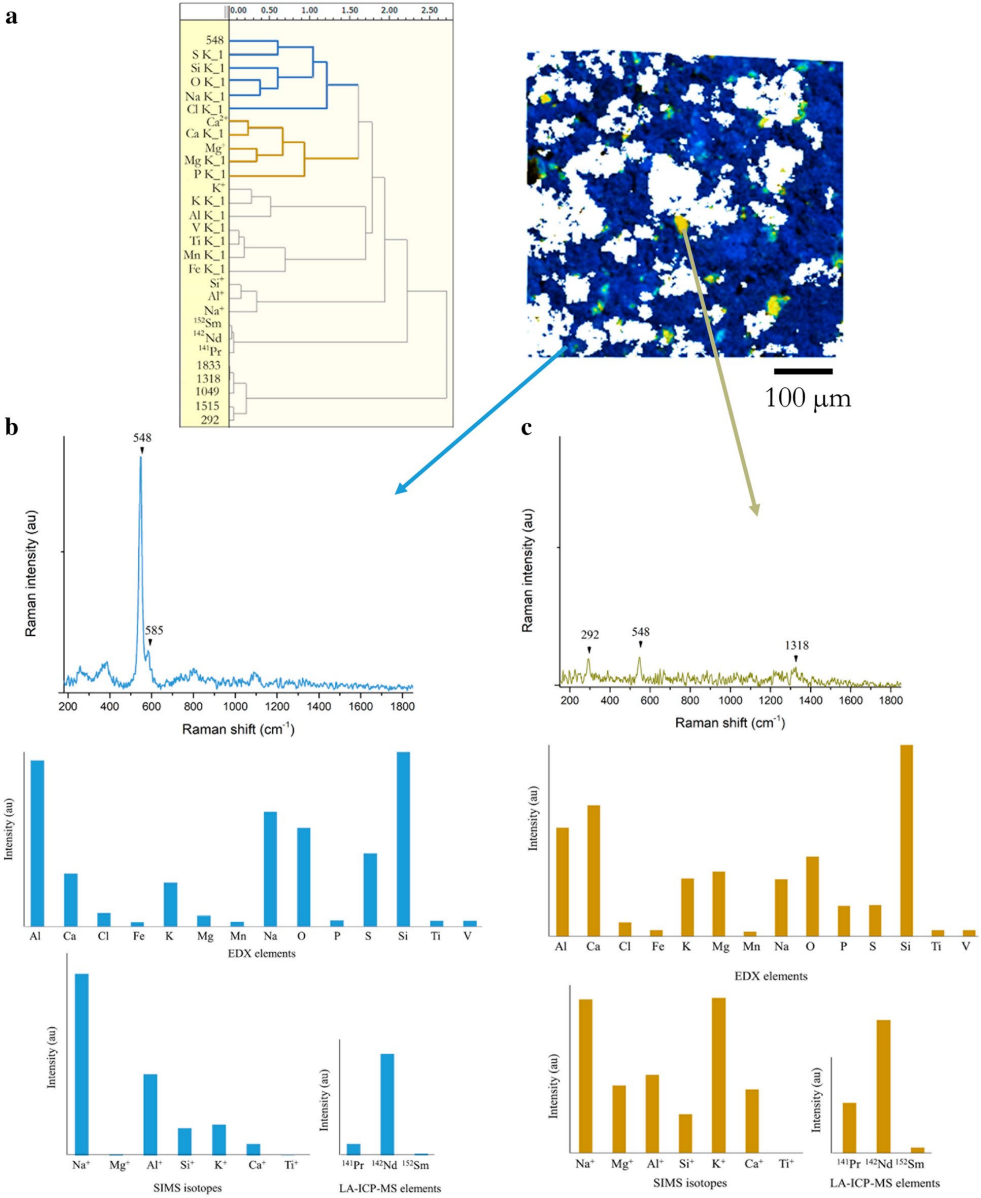
(**a**) Dendrogram of the loadings of the first five principal components of NU-1 sample PCA model, and SEM image superposed with the spatial distribution of the two sub-clusters marked in blue and dark yellow. Representative Raman spectra, EDX, SIMS, and LA-ICP-MS of blue (**b**) and dark yellow (**c**) sub-clusters.

For this discussion, we will focus on the natural pigments as they are more complex due to their higher heterogeneity. PCA analysis performed on the defined SPDCs for the different pigments and followed by an HCA of the loadings significantly differentiates several sub-clusters. The low percentage of variance captured by the first five PCs in all the models for the different samples (in the order of 10–16% each) reveals the high complexity of the dataset. The need for scaling to merge data from the different techniques (with different units and measurement ranges) can also contribute to this behaviour. Despite this fact, useful information can be extracted from the MSHSI datacube. In Fig. 6, the dendrogram obtained from the purified pigment (NU-1) is shown as an example. The most distinct sub-cluster concentrates all the bands in the region 1200–1850 cm^−1^ of the Raman spectrum (attributed to luminescence effects in previous studies) as well as the bands at 292 and 1049 cm^−1^. The three REE elements (Nd, Pr, and Sm) also form a distinctive sub-cluster, which indicates a similar spatial distribution of Nd, Pr, and Sm. The rest of SPDCs can be attributed to silicate mineral phases with several sub-clusters. One of the sub-clusters (blue in Fig. 6a) contains the Raman band at 548 cm^−1^ and the EDX Kα X-ray emissions of Si, Na, and O. Additionally, a sub-cluster containing EDX and SIMS descriptors of Mg and Ca elements is observed (dark yellow in Fig. 6a). In Fig. 6, the superposition of the SEM image and the images of these two sub-clusters from PCA-HCA of the MSHSI datacube are depicted together with the extracted mean spectra.

The MSHSI datacube is showing its effectiveness in locating the most abundant mineral phase in the purest pigment (NU-1). The high relative intensities of Na, Si, O, and S elements correlate well with the strong Raman band at 548 cm^−1^, which dominates the Raman spectrum of lazurite. As explained before, this Raman feature is attributed to stretching vibrations of S_3_^−^ radical trapped in lazurite β-cages. It is, therefore, reasonable to assume that this cluster is indicating the distribution of this mineral (Na_8_Al_6_Si_6_O_24_S_n_) in the sample (Fig. 6b). On the other hand, the second sub-cluster (Fig. 6c) could be attributed to diopside mineral (CaMgSi_2_O_6_), a common impurity present in natural ultramarine pigments^40^. However, the Raman spectra registered in these areas are very weak and do not present significant features apart from a small contribution from lazurite. This fact hinders unambiguous identification of any mineral phase. Another sub-cluster contains the SIMS mass of 38.94 m/z (K) and EDX Kα X-ray emissions of K and Al, which probably corresponds to disperse KBr particles that have not been fully eliminated with the KBr mask. The others sub-clusters cannot be attributed to specific chemical species. However, they reveal some tendencies in the data, for example, it seems that transition metal impurities (V, Mn, Ti, Fe) are distributed within the different aluminosilicate minerals without concentrating in a certain mineral phase.

Similar results were obtained for NU-2 and SU samples. However, when analyzing the lowest quality natural pigment (NU-3) and the by-product of the purification process (NU-A), the Raman band at 548 cm^−1^ did not form such a distinct cluster with Na, Al, Si, O, and S. This is consistent with the fact that these low-quality pigments macroscopically do not display a bright blue tone and few blue areas can be detected when observing them at the microscopic scale. The presence of accessory minerals in the chemical composition of the natural pigment is very common, as previously reported in literature^37^. Apart from the already discussed pyrite and diopside, lazurite can be found in the company of calcite (CaCO_3_), forsterite (Mg_2_SiO_4_), phlogopite (K(Mg, Fe, Mn)_3_Si_3_AlO_16_(F(OH)_2_)^40^, nosean (Na_8_Si_6_Al_6_O_24_(SO_4_)·H_2_O)^42^ or haüyne (Na_3_CaSi_3_Al_3_O_12_(SO_4_))^43^, among others. Most of the aforementioned impurities have an aluminosilicate base, together with a few other elements, such as Na, Ca, O, S, Mg, Fe, or Cl. For this reason, the association of several of these elements in clusters is expected and the identification of different mineral phases based on the elemental composition is challenging.

**Figure 7:**
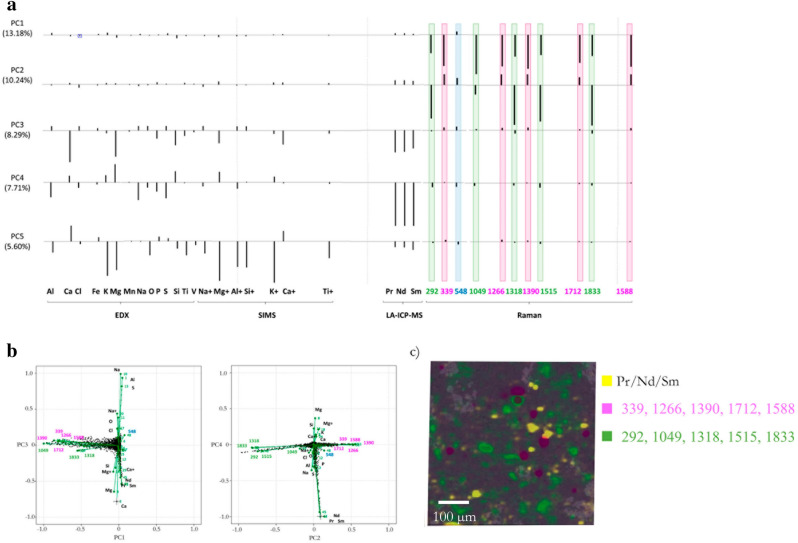
(**a**) Loadings of PC1 to PC5 obtained in NU-3 sample PCA model. (**b**) Scores-loadings biplot of PC1 vs PC3 and PC2 vs PC4. (**c**) SEM image superposed with the spatial distribution of clusters corresponding to REE elements (yellow) and characteristic Raman bands (pink and green).

In fact, the most interesting feature observed in low-quality natural samples is the presence of bands in the 1200–1850 cm^−1^ region of the Raman spectra. In Fig. 7, which shows the PCA results for the NU-3 sample, it can be seen that these Raman bands form the most important contributions for PC1 and PC2. Looking at the bi-plots (see Fig. 7b), PC1 clearly distinguishes between areas showing bands in the 1200–1850 cm^−1^ region (with negative values for PC1) and the rest of the sample (with values close to 0). On the other hand, PC2 allowed the distinction between bands of different chemical origin, as can be seen in the biplot of PC2 vs PC4, where they show a negative correlation. Raman bands at 1266, 1390, 1588, and 1712 clustered together with 339 cm^−1^ and are pointing to positive PC2 values, whereas 1318, 1515, 1833 together with 292 cm^−1^ showed negative PC2 values. The two groups of Raman bands can be identified as the endmembers c and b, respectively, identified by VCA.

As mentioned before, the origin of these bands remains unclear, although Schmidt^37^ attributed them to luminescence effects due to the presence of transition metals like Ti. However, this assumption was based on quantitative analysis of bulk samples. In this work, the investigation of the spatial distribution of trace elements, like transition metals and rare earth elements (REE) can aid to elucidate their origin. From our results, it is clear that these bands do not show a clear grouping with either a transition metal or REE. Furthermore, they cannot be associated with diopside impurities, as it was also suggested by Schmidt and in our previous work, since the elements typically related to this mineral (Mg, Ca, and Si) are arranged perpendicular to these Raman features in both biplots (see Fig. 7b). In fact, no clear correlation was found between these features and any of the chemical elements detected with good signal to noise ratio.. On the other hand, PC3 allocates a certain correlation between REE (Nd, Pr, and Sm) and diopside characteristic elements, while this correlation is negative for PC4. Thus, REE must also be distributed within different mineral phases as shown before for transition metals.

**Figure 8:**
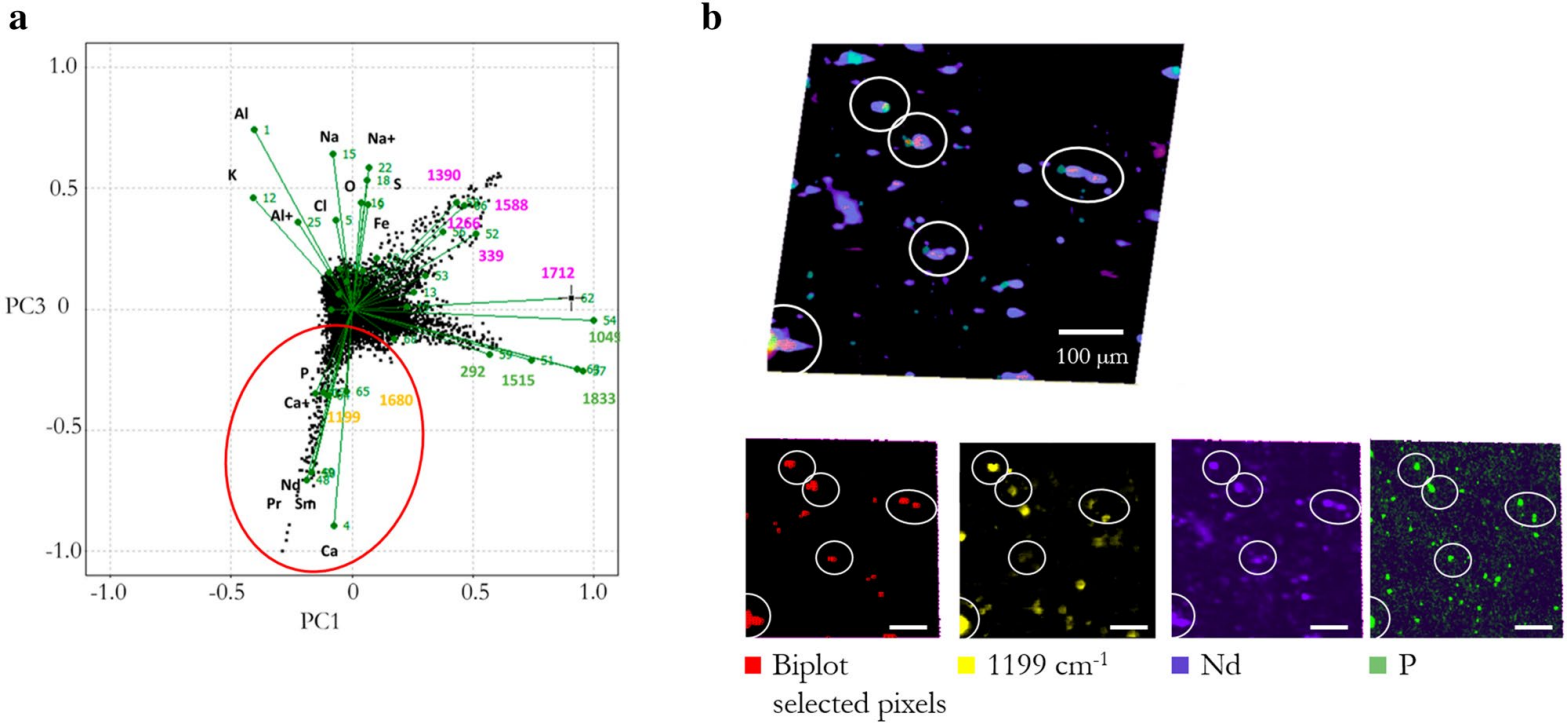
(**a**) PC1 vs PC3 scores-loading biplot of NU-A sample and (**b**) superposition of the spatial distribution of selected pixels (red ellipse in a), Nd, P, and 1199 cm^−1^ Raman band. Individual distributions are included for better visualization. Main areas of coincidence are marked in white. Scale bar corresponds to 100 μm.

Finally, in the case of the ultramarine ash sample (NU-A), an additional endmember was identified when analyzing the Raman sub-dataset (endmember d). Interestingly, in this case, a clear correlation was found (see Fig. 8a) between its characteristic bands at 1199 and 1680 cm^−1^ and Ca, P, and REE elements. However, the spatial distribution of Ca, P, and REE (all showing very similar distribution), does not match. In particular, Ca is much more widespread in the sample. Nevertheless, the interesting finding is that there some spots where these elements coincide and the bands at 1199 and 1680 cm^−1^ also appear as can be seen Fig. 8b. This effect could be attributed to luminescence of apatite [Ca_5_(PO_4_)_3_(F, OH, Cl)], a minor but ubiquitously present mineral in diverse terrestrial rocks, hosting REE elements, in particular neodymium. Nd^3+^ has both the ionic radius and minimal charge difference for ease of substitution of calcium and the required energy-level arrangement for 785 nm excited fluorescence, which would explain the widespread occurrence of fluorescence in this region of the Raman spectra of calcium-based minerals, as reported in different studies^44,45^.

### On the origin of the bands in the region 1200–1850 cm^−1^

Considering the above-mentioned results, we suggest an alternative hypothesis for the origin of the rest of the bands: the presence of CO_2_ trapped in the β-cages of the sodalite-type structure. The Raman spectrum of CO_2_ is characterized by two bands at ca. 1285 cm^−1^ and 1388 cm^−1^, usually called Fermi diad, with the Raman band at higher wavenumbers being more intense. They correspond to the symmetric-stretching fundamental (ν1) and the bending first overtone (2ν2), which accidentally degenerate with the same symmetry^46,47^. These frequencies are in good agreement with the bands observed here for the endmember c at 1266 and 1390 cm^−1^. For the endmember b, the most abundant one, these bands are shifted to lower frequency (1244 and 1318 cm^−1^) with the shift of the upper Fermi resonance band being more pronounced. This behavior is expected to occur upon coordination since the accompanying change in electron density induces symmetry breaking of the linear molecule^48^. Thus, we can attribute these two endmembers to trapped CO_2_ molecules, weakly and more strongly bonded to the aluminosilicate network, respectively. In addition, the band at 1049 cm^−1^ present in the Raman spectra of both endmembers could be attributed to the formation of CO_3_^2−^ ions^48,49^ and the bands observed at 292 and 339 cm^−1^ (for endmembers b and c, respectively) to low-frequency lattice modes of the aluminosilicate network hosting the CO_2_ molecules^49^. The question that arises is why these Raman features between 1200 and 1400 cm^−1^ have been observed only with excitation at 785 nm. The reason is that when using either 532 nm or 633 nm the strong resonance effect of the S_3_^−^ chromosphere hinders the observation of any other spectral features. Our hypothesis regarding CO_2_ presence is also supported by several studies performed using FTIR on lapis lazuli pigments and lazurite-type minerals^49–51^. Smith and Klinshaw^51^ reported the presence of CO_2_ in Kremer pigment’s natural crushed and washed Afghan lapis lazuli samples (NU-2 and NU-3) as revealed by its characteristic IR band at 2340 cm^−1^. The purified pigment (NU-1) on the contrary showed only a weak CO_2_ signature. This fact was attributed to the purification process that most likely has reduced the amount of CO_2_ in this pigment. Such findings are in good agreement with the behavior of the Raman bands observed here in the region 1200–1400 cm^−1^. Furthermore, Bellatreccia et al.^52^ studied a large set of specimens of the haüyne-nosean group showing that these minerals are systematically very rich in structural carbon dioxide. Their thermal behavior also supports the claim for a strong interaction between CO_2_ and the aluminosilicate framework: upon heating, there is a complete and irreversible loss of water, whereas CO_2_ is retained up to temperatures higher than 900 °C.

## Conclusions

In the presented study, the successful application of using a multisensor hyperspectral imaging approach for the characterization of natural and synthetic ultramarine pigments has been demonstrated. The complementary information of hyperspectral data acquired using four different analytical techniques, providing both elemental and molecular information, has been combined. In this respect, MSHSI has been proven to be a versatile methodology to better understand samples as complex as historical pigments of complex mineral composition. Microanalytical techniques for trace analysis have provided a detailed elemental fingerprint of the samples. We have confirmed the presence of impurities as pyrite mineral in natural pigments with the appearance of a Fe–S cluster in the analysis of low-quality samples. No correlation between the distribution of the supposed luminescence bands and the transition metals (Fe, Mn, Cr, V, Ti) or the REE (Nd, Pr, Sm) detected was found. Also, they could not be associated with the cluster formed by Ca, Mg and Si, hence, the hypothesis of the association of these bands with diopside (CaMgSi_2_O_6_) impurities must be also disregarded. Considering these results, an alternative hypothesis on their origin has been proposed which we believe is strongly supported by our experimental data and careful analysis made possible by the chosen multi-sensor approach and stepwise data analysis. Although further studies are certainly needed to fully corroborate the explanation of these Raman features, our study definitively shows that all the previous hypotheses based on bulk analyses lead to wrong conclusions, revealing the importance of multi analytical chemical imaging approaches.

## Materials and methods

### Samples and sample preparation

Ultramarine blue pigments were purchased in powder form Kremer Pigmente GmbH & Co. KG (Germany) (Table 2). Pellets of each pigment were prepared using a hydraulic press after mixing the powder with KBr of spectroscopic grade in a 1:1 ratio.

**Table 2:** Natural and synthetic ultramarine blue pigments employed in this study.

Sample code	Kremer name	Kremer reference
NU-1	Purified *lapis lazuli*	10530.12010.315
NU-2	*Lapis lazuli,* good quality	10520.12010.104
NU-3	*Lapis lazuli,* medium quality	10510.12010.104
NU-A	Ultramarine ash	10580.12050.104
SU	Ultramarine blue, dark	45010.12100.136

### Instrumentation and measurements

Raman analysis was carried out using a WITec alpha300 RSA + confocal microscope (WITec GmbH, Ulm, Germany). For the measurements, a 785 nm laser source set to 10mW and a 10 × objective (Zeiss, 0.25 NA) were used. A total sample area of 500 µm × 500 µm^2^ with 250 × 250 pixels (pixel size of 2 µm) was analyzed for each pigment. The scattered photons were collected in backscattering mode and detected using a fiber-coupled spectrometer (UHTS 3600, f/4, 300 mm focal length) equipped with a 600 g/mm grating and a deep depletion charged couple device (DD CCD; Andor Technology Ltd., Belfast, UK). The acquisition time was set to 1 s per spectrum.

SEM–EDX measurements were performed using an electron microscope Quanta 200 (Thermo Fisher Scientific FEI Europe BV, Eindhoven) coupled to an EDAX spectroscope (AMETEK GmbH EDAX Business Unit). Employing a 500 × magnification, a total area of 597 µm × 500 µm^2^ was investigated. Acceleration voltage were set to 20 kV to ensure the detection of Fe (Fe K_α_ ≈ 6.4 keV) without interferences. Spatial resolution for SEM images was 50 nm and for EDX measurements the pixel size was ≈ 1.2 µm. EDX images were accumulated to achieve reasonable signal-to-noise ratios of the elemental distributions.

For the SIMS analysis, a ToF-SIMS^5^ spectrometer (IONTOF GmbH, Münster, Germany) equipped with a 25 keV bismuth liquid metal ion gun (LMIG) was employed. Data were obtained by operating in positive-ion mode and a gentle oxygen sputtering process of 3 s (crater size 1000 µm × 1000 µm^2^ with 2 kV O_2_ gun) was carried out before the measurements to activate the surface and enhance the signal of the positive ions. First, a mass spectrum was collected from within the marked sample area before images of the sample area were collected using the high lateral resolution mode. The primary ion beam settings were as follows: region of interest 500 µm × 500 µm^2^, Bi^1+^, 25 keV, raster mode saw tooth, raster size 1024 by 1024 pixels, and 32 scans. The isotope with the most intense signal for each element was selected for the analysis.

Finally, LA-ICP-MS measurements were carried out using an ESI NWR213 (Fremont, CA) laser ablation system operating at a wavelength of 213 nm coupled to an iCAP Qc ICP-MS system (ThermoFisher Scientific, Bremen, Germany) using PTFE tubing. The samples were ablated under a constant stream of helium (0.65 L/min). Argon was used as a make-up gas (1 L/min) before introducing the aerosol to the ICP-MS. Tuning of the instrument was carried out daily for maximum ^115^In signal using a NIST612 glass standard (National Institute of Standards and Technology, Gaithersburg, MD). Data was collected using Qtegra 2.10 provided by the manufacturer of the instrument. Each sample was analysed using a laser spot size of 8 µm balancing spatial resolution and sensitivity. 100 parallel line scans with a length of 1000 µm each and a distance of 8 µm between each line were measured with a scan speed of 16 µm/s on each sample resulting in a total analyzed area of 1000 µm × 800 µm^2^ with a lateral resolution of 8 µm. The laser was operated with 20 Hz repetition rate and a fluence of 11.9 J/cm^2^. Ablation rate of LA-ICP-MS imaging experiments were determined using a profilometer (DektakXT, Bruker, Massachusetts, USA). Preliminary bulk analyses considering 11 rare earth elements (Pr, Nd, Eu, Sm, Gd, Tb, Dy, Ho, Er, Tm and Yb) were performed. In order to get the highest lateral resolution possible, while ensuring sufficient signal intensity, only those elements showing the largest intensity levels were selected for imaging purposes: ^141^Pr, ^142^Nd and ^152^Sm. Additionally, ^27^Al and ^39^K were monitored. For each isotope, the dwell time of the ICP-MS was set to 10 ms.

### Multimode hyperspectral imaging data fusion and data analysis

ImageLab (Release 3.45, Epina GmbH, Austria)^53^ software package was used for spectral processing, HSI datasets-fusion, and data analysis. To reduce the dimensionality of the data, pre-defined spectral descriptors (SPDCs; see section Preliminary analysis) were selected. Signal intensities corresponding to the different elements were used for data analysis in the case of EDX, LA-ICP-MS, and SIMS datasets. Raman spectra (spectral range 100–1900 cm^−1^) were first preprocessed by spike removal and baseline correction (by Eilers Algorithm^54^ with 7 iterations) employing ImageLab software. Raman spectral descriptors consisted of areas of fifteen bands selected according to the results of Vertex Component Analysis (VCA) (see section “Preliminary analysis”). Principal component analysis (PCA) was performed on the MSHSI datacube, after standardization. Standardization mean-centers the data and then divides the individual variables by their respective standard deviation. Furthermore, hierarchical cluster analysis (HCA) was performed on the loadings of the PCs selected by considering both the cumulative explained variance and the inter and intra-cluster distances.

## Supplementary Information

Supplementary Information.
