# Supplementary material for: Multisensor hyperspectral imaging approach for the microchemical analysis of ultramarine blue pigments
Source: Sci Rep. 2022 Jan 13;12:707. doi: 10.1038/s41598-021-04597-7 (PMC8758711; doi:10.1038/s41598-021-04597-7)
Supplement: Supplementary file 1 — Supplementary Information. [file 41598_2021_4597_MOESM1_ESM.pdf]

Manuscript: "Multisensor hyperspectral imaging approach for the microchemical analysis of ultramarine blue pigments"

Authors: González-Cabrera, M., Wieland. K., Eitenberger, E., Bleier, A.,Brunnbauer, L., Limbeck, A., Hutter, H., Haisch, C.,Lendl, B., Domínguez-Vidal, A., Ayora-Cañada. M.J.*

Supplementary information. Figures

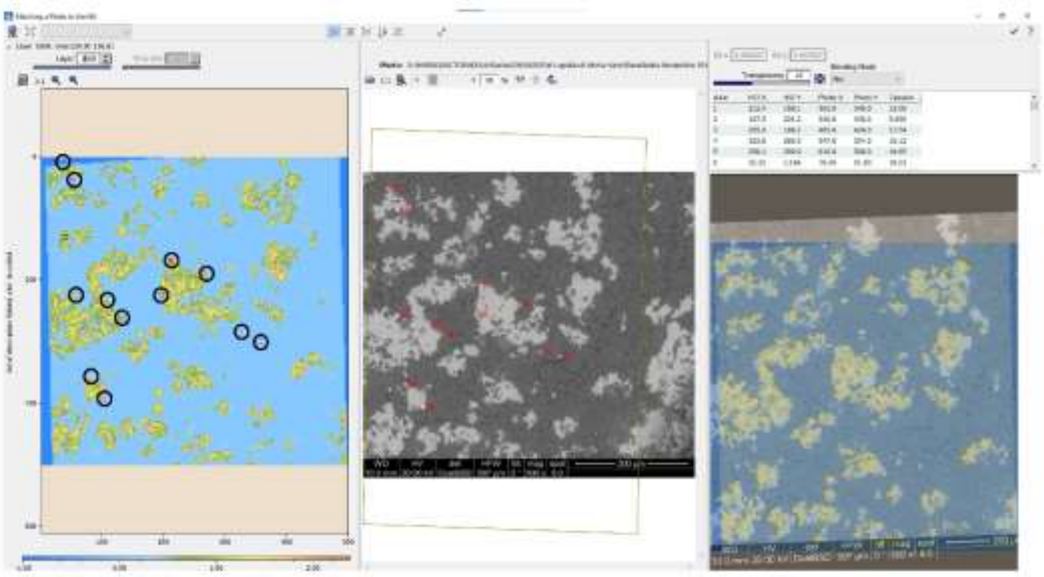

Figure S1. Screenshot taken from ImageLab software showing the image alignment process

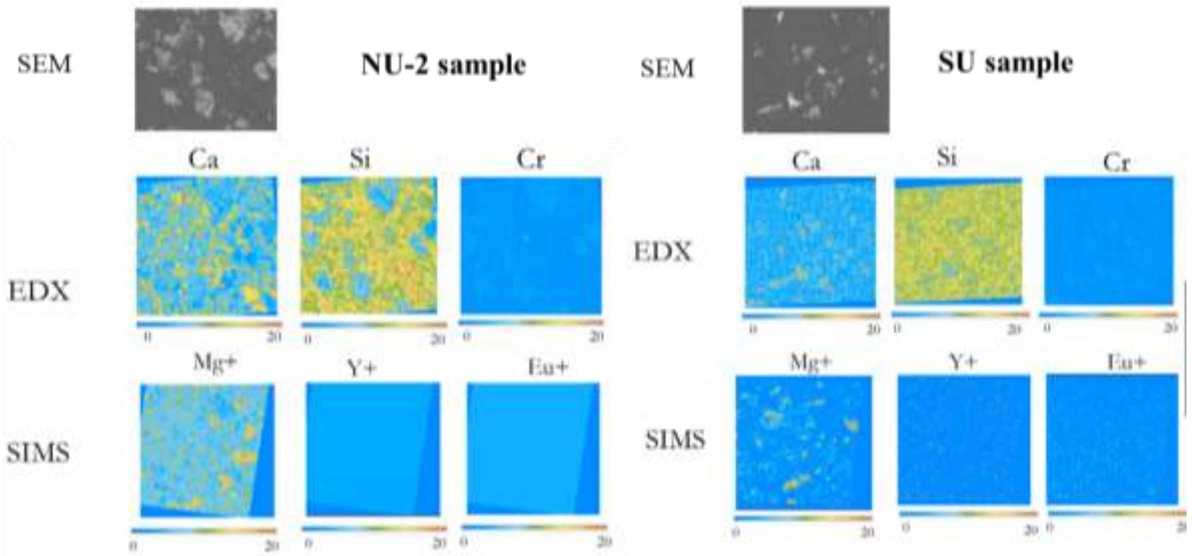

Figure S2. Spatial distribution of Ca, Si and Cr (EDX) and Mg+, Y+ and Eu+ (SIMS) for the NU-2 and SU samples. Corresponding SEM image is also included.

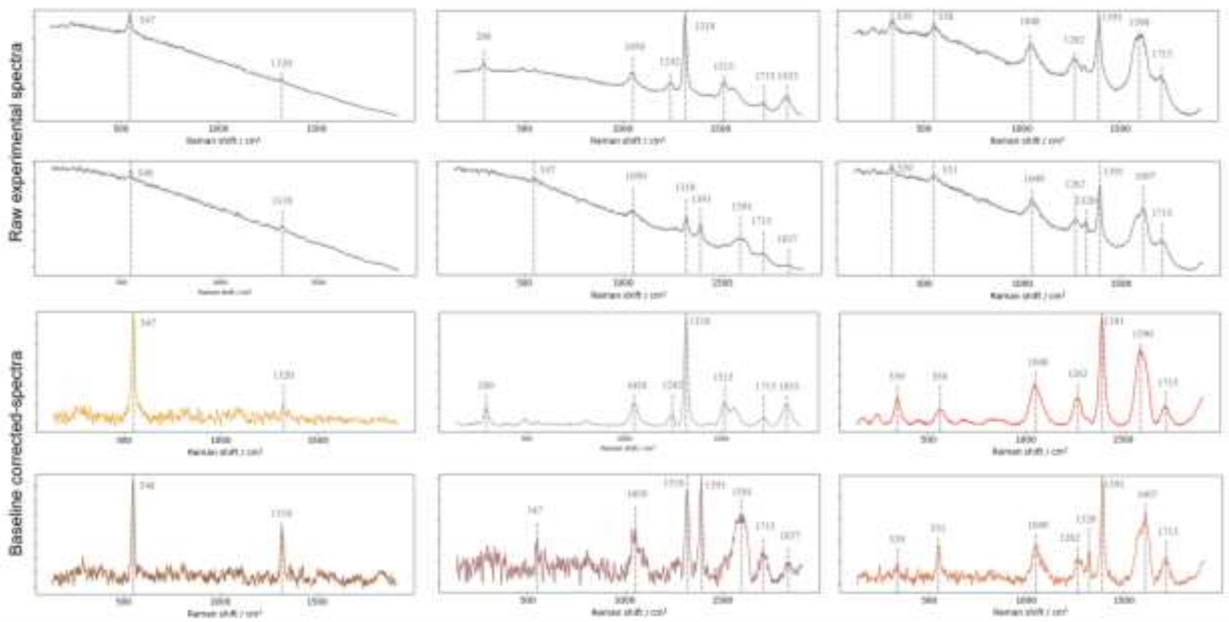

Figure S3. Examples of experimental Raman spectra (raw and baseline-corrected)
